# Connecting lysosomes and mitochondria – a novel role for lipid metabolism in cancer cell death

**DOI:** 10.1186/s12964-019-0399-2

**Published:** 2019-07-29

**Authors:** Karin Bartel, Helmut Pein, Bastian Popper, Sabine Schmitt, Sudha Janaki-Raman, Almut Schulze, Florian Lengauer, Andreas Koeberle, Oliver Werz, Hans Zischka, Rolf Müller, Angelika M. Vollmar, Karin von Schwarzenberg

**Affiliations:** 1 Department of Pharmacy, Pharmaceutical Biology, Ludwig-Maximilians-Universität München, Butenandtstr. 5-13, 81377 Munich, Germany; 2 Chair of Pharmaceutical/Medicinal Chemistry, Institute of Pharmacy, Friedrich Schiller University Jena, Philosophenweg 14, 07743 Jena, Germany; 3 Department of Anatomy and Cell Biology, Biomedical Center, Ludwig-Maximilians-Universität München, Grosshaderner Strasse 9, 82152 Planegg-Martinsried, Germany; 4 Institute of Toxicology and Environmental Hygiene, Technical University Munich, School of Medicine, 80802 Munich, Germany; 5 Department of Biochemistry and Molecular Biology, Theodor-Boveri-Institute, Biocenter, Am Hubland, 97074 Würzburg, Germany; 6 Institute of Molecular Toxicology and Pharmacology, Helmholtz Center Munich, German Research Center for Environmental Health, 85764 Neuherberg, Germany; 7 Helmholtz Centre for Infection Research and Department of Pharmaceutical Biotechnology, Helmholtz Institute for Pharmaceutical Research Saarland, Saarland University, PO 151150, Universitätscampus E8 1, 66123 Saarbrücken, Germany

**Keywords:** Lysosome, V-ATPase, Mitochondria, Fission, Apoptosis, Lipid metabolism, Cardiolipin

## Abstract

**Background:**

The understanding of lysosomes has been expanded in recent research way beyond their view as cellular trash can. Lysosomes are pivotal in regulating metabolism, endocytosis and autophagy and are implicated in cancer. Recently it was discovered that the lysosomal V-ATPase, which is known to induce apoptosis, interferes with lipid metabolism in cancer, yet the interplay between these organelles is poorly understood.

**Methods:**

LC-MS/MS analysis was performed to investigate lipid distribution in cells. Cell survival and signaling pathways were analyzed by means of cell biological methods (qPCR, Western Blot, flow cytometry, CellTiter-Blue). Mitochondrial structure was analyzed by confocal imaging and electron microscopy, their function was determined by flow cytometry and seahorse measurements.

**Results:**

Our data reveal that interfering with lysosomal function changes composition and subcellular localization of triacylglycerids accompanied by an upregulation of PGC1α and PPARα expression, master regulators of energy and lipid metabolism. Furthermore, cardiolipin content is reduced driving mitochondria into fission, accompanied by a loss of membrane potential and reduction in oxidative capacity, which leads to a deregulation in cellular ROS and induction of mitochondria-driven apoptosis. Additionally, cells undergo a metabolic shift to glutamine dependency, correlated with the fission phenotype and sensitivity to lysosomal inhibition, most prominent in Ras mutated cells.

**Conclusion:**

This study sheds mechanistic light on a largely uninvestigated triangle between lysosomes, lipid metabolism and mitochondrial function. Insight into this organelle crosstalk increases our understanding of mitochondria-driven cell death. Our findings furthermore provide a first hint on a connection of Ras pathway mutations and sensitivity towards lysosomal inhibitors.

**Graphical Abstract:**

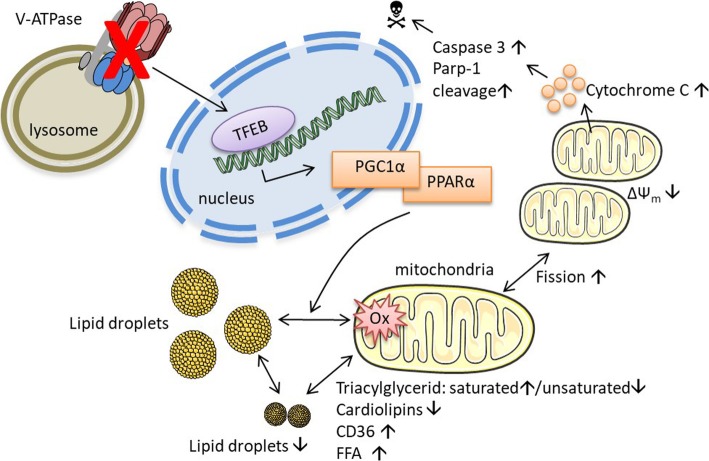

**Electronic supplementary material:**

The online version of this article (10.1186/s12964-019-0399-2) contains supplementary material, which is available to authorized users.

## Background

Historically the lysosome has simply been regarded as the recycling compartment of a cell, yet recent research identified the lysosome as pivotal in regulating cellular metabolism [1]. Lysosomes are small organelles with an acidic interior, which host a large number of hydrolytic enzymes like proteases, lipases and nucleases. These hydrolases are responsible for degradation and recycling of macromolecules or even whole organelles thereby regulating endocytosis and autophagy [2]. Lysosome function or malfunction was found to play an important role in different diseases including cancer [3]. Interestingly, tumor cells often have an increased lysosomal activity and autophagy level as compared to non-malignant cells strengthening the hypothesis of the importance of lysosomes in resisting energetic stress conditions [4]. Especially the recent discovery of its key role in lipid metabolism highlights it as a promising organelle in regard of cancer treatment.

Cellular lipid and cholesterol metabolism has emerged as novel target for cancer treatment, as it is frequently deregulated in tumor tissue. Yet, mechanistic data on how influencing lipid metabolism limits cancer cell survival is still limited [5, 6]. While there are many descriptive studies showing alterations in cancer cell lipid metabolism as compared to healthy tissue and several studies link deregulation in lipid metabolism to malignancy, the influence of specific alterations on cellular organelles has not been thoroughly investigated [7, 8]. Recently, we were able to identify a crucial role of the lysosome in cholesterol metabolism. Targeting the lysosomal V-ATPase, a proton pump necessary for lysosomal acidification, has been shown to restrict intracellular cholesterol availability and thereby inhibits tumor progression, linking lipid metabolism to lysosomes [9]. Targeting the V-ATPase with various natural compounds, such as archazolid, bafilomycin, concanamycin or iejimalide, has also been discovered to induce mitochondria-driven apoptosis in different cancer cells and to modulate autophagy, however the detailed mechanisms leading to mitochondrial apoptosis induction remain undiscovered [10–13].

The role of mitochondria in cell death induction has been studied extensively in the past. Their pivotal role in releasing proteins from the inter-membrane space to the cytosol and their power to activate caspases has long been known. Interestingly though interaction of mitochondria with other organelles has been less investigated [14–16]. In the present study we discovered a novel connection between lysosomes and mitochondria, in which cellular lipid metabolism plays an essential role. We provide a novel mechanistic insight into a triangle of lysosomes, mitochondria and lipid metabolism, shedding light on a missing link in lysosomal induced, mitochondria-driven cell death.

## Methods

### Compounds and cell culture

HUH7 were obtained from JCRB, BxPC3 and Panc03.27 were obtained from ATCC. STR profiling and routine testing for mycoplasma contamination were performed. HUH7 were grown in DMEM, 10% FCS, BxPC3 were grown in RPMI-1640, 10% FCS and Panc03.27 were grown in RPMI-1640, 15% FCS, 10 Units/ml human recombinant insulin (PAN-Biotech GmbH, Aidenbach, Germany & Sigma-Aldrich). All cells were cultured under constant humidity at 37 °C, 5% CO_2_. For HUH7, all plastic ware was pre-coated with 0.001% collagen G (PBS). Archazolid A was provided by Rolf Müller, Torin1, CCCP, BPTES, UK5099 and Etomoxir were purchased from Sigma-Aldrich, and dissolved in DMSO (Sigma-Aldrich).

### Triaclyglycerid (TAG) and acyl-CoA analysis

HUH7, HepG2 or Hep3 cells were treated as indicated and collected by centrifugation. Lyososomes [17] or mitochondria were isolated as described previously [18], or whole cells were used. Cell pellets and subcellular fractions were frozen in liquid nitrogen and stored at − 80 °C until use. TAGs were extracted using a mixture of PBS pH 7.4, methanol, chloroform, and saline (14:34:35:17), separated on an AcquityTM UPLC BEH C8 column (1.7 μm, 2.1 × 100 mm, Waters, Milford, MA) using an AcquityTM Ultraperformance LC system (Waters) and detected by a QTRAP 5500 mass spectrometer (Sciex, Darmstadt, Germany) equipped with an electrospray ionization source as described [19]. Acyl-CoAs were extracted with methanol/water (70/30), separated on an AcquityTM UPLC BEH C18 column (1.7 μM, 2.1 × 50 mm), and analyzed in the positive ion mode based on the neutral loss of 2′-phospho-ADP ([M + H-507]+) as previously reported for malonyl-CoA [20]. 1,2-Dimyristoyl-sn-glycero-3-phosphatidylcholine and [13C3]-malonyl-CoA were used as internal standards for TAGs and acyl-CoAs, respectively.

### Confocal microscopy

30,000 cells/well were seeded on IBIDI μ-slides (IBIDI, Martinsried, Germany) one day before treatment as indicated (24 h). For antibody staining, cells were washed (PBS), fixed (3% Paraformaldehyde) for 30 min, permeabilized (0.1% Triton-X) and unspecific binding was blocked (5% BSA) after treatment. Primary antibodies (Lamp3/sc-15363, Hsp60/sc-1052, Cox4/4844 NEB, ACADVL/ab118183, ACADM/ab118183, HADHA/ab118183, TFE3/PA5–35210, MITF/sc-71588, TFEB/A303-673A) were applied over night at 4 °C, secondary antibodies (Alexa-Fluor 488/A-11008, Alexa-Fluor 546/A-10040, Alexa-Fluor 633/A-21082) and Hoechst33342 for 45 min at 25 °C. LD were stained by 2 μM Bodipy™ 493/503 for 30 min, fatty acids were tracked by adding 1 μM BODIPY™ 558/568 C_12_ (both: Thermo Fisher) 16 h prior to end of treatment. Cells were washed, mounted with FluorSave™ Reagent (Beckman Coulter) and covered with a glass coverslip. For life cell imaging 2 μM Bodipy™ 493/503 was added 30 min prior to imaging, 1 μM BODIPY™ 558/568 C_12_ was added 16 h prior to imaging, 100 nM LysoTracker™ Blue DND-22 or 100 nM MitoTracker™ Green FM were added for 30 min to visualize lysosomes or mitochondria. Medium was exchanged and images were acquired using a Leica TCS SP 8 SMD confocal microscope (Leica TCS SP 8 SMD, Wetzlar, Germany) with a top stage incubator (Oko Lab, Ottaviano, Italy).

### Flow cytometry

For surface expression cells were treated as indicated (24 h), harvested and stained with specific antibody against CD 36 (sc-5522) and fluorescent secondary antibody Alexa-Fluor 546 (A-11056). Mitochondrial mass was detected after labelling mitochondria (MitoTracker™ Green FM) for 30 min prior to the end of treatment time. For analysis of mitochondrial membrane potential cells were loaded with DIOC6 (Sigma Aldrich) or JC-1 (Sigma Aldrich). Enzyme abundance of FAO enzymes was detected according to manufacturer’s protocol (Abcam, ab118183). Mitochondrial superoxide and cellular ROS were detected by loading the cells with MitoSOX™ (M36008, Thermo Fisher) or CDCFDA (C1165, Thermo Fisher) prior to harvesting. Cytosolic cytochrome C was detected by specific antibody (NEB, 4272) and fluorescent secondary antibody Alexa-Fluor 546 (A-11056). Subdiploid DNA content was determined according to Nicoletti et al. [15]. Briefly, cells were treated as indicated, harvested, permeabilized with sodiumcitrate containing Triton X-100, stained with 25 μg/ml propidiumiodide and analyzed. Subdiploid cells left of the G1-peak were considered as apoptotic. All cells were analyzed by flow cytometry (Canto II, Beckton Dickinson, Heidelberg, Germany).

### Quantitative real-time PCR

Total mRNA was isolated from cell culture samples according to manufacturer using Qiagen RNeasy Mini Kit (Qiagen, Hilden, Germany). For cDNA synthesis, the High Capacity cDNA Rerverse Transcription Kit (Applied Biosystems, Foster City, CA) was used. qRT-PCR was performed with the QuantStudio™ 3 System (Thermo Fisher) and the SYBR Green PCR Master Mix (Thermo Fisher) according to the manufacturer’s instructions. All designed primers were purchased from Metabion (Martinsried, Germany).

### Western blot

For preparation of whole cell lysate, cells were collected by centrifugation, washed with ice-cold PBS, and lysed for 30 min in 1% Triton X-100, 137 mM NaCl, and 20 mM Tris-Base (pH 7.5) containing the protease inhibitor complete (Roche). Lysates were centrifuged at 10,000 g/10 min at 4 °C. Mitochondria were isolated as described previously [10]. Equal amounts of protein were separated by SDS-PAGE and transferred to nitrocellulose membranes (Hybond-ECLTM, Amersham Bioscience). Membranes were blocked with 5% BSA in PBS containing 0.1% Tween 20 for 2 h and incubated with specific antibodies against PGC1α/ab54481, PPARα/MA1–822, Mitofusin-1/14739 NEB, Drp-1/8570 NEB, pDrp-1 Ser 637/6319 NEB, pDrp-1 Ser 616/4494 NEB, ACADVL/ab118183, ACADM/ab118183, HADHA/ab118183, TFE3/PA5–35210, MITF/sc-71588, TFEB/A303-673A, Bax/sc-493, Bak/ab32371) over night at 4 °C. Proteins were visualized by secondary antibodies conjugated to horseradish peroxidase (HRP) and freshly prepared ECL solution, containing 2.5 mM luminol. Chemiluminescence signal was detected with the ChemiDoc™ Touch Imaging System (Bio-Rad, Munich, Germany).

### Analysis of free fatty acids

Free fatty acids were detected according to manufacturer’s protocol (MAK044, Sigma Aldrich). Briefly, cells were treated as indicated, harvested and homogenized in 1% Triton X-100 in chloroform. Organic phase was collected after centrifugation and vacuum dried. Lipids were re-dissolved in assay buffer and incubated with reaction mix. Absorbance was measured with an infinite F200Pro plate reader (Tecan) and is proportional to free fatty acid content.

### Analysis of cardiolipins

Detection of cardiolipins in cell lysates or in isolated mitochondria was performed according to manufacturer’s protocol (#K944–100, BioVision). Briefly, cells were treated as indicated, harvested and lysed. Lysate was loaded with a CL-Probe and incubated for 10 min at 25 °C. Probe fluorescence was recorded at Ex/Em 304/480 nm with an infinite F200Pro plate reader (Tecan) and is proportional to cardiolipin content.

### Electron microscopy

Samples were fixed with 2.5% Glutaraldehyde in 0.1 M Sodium Cacodylate Buffer, pH 7.4 for 24 h at the minimum. Glutaraldehyde was removed, samples were washed 3x with 0.1 M Sodium Cacodylate Buffer, pH 7.4. Postfixation and prestaining was done for 45- 60 min with 1% osmium tetroxide, ddH2O, 3.4% NaCl and 4.46% potassium dichromate pH 7.2. Samples were washed 3x with ddH2O and dehydrated with an ascending ethanol series (15 min with 30, 50, 70, 90 and 96%, respectively and 2 × 10 min with 100%) and propylene oxide (2 × 30 min). Subsequently, samples were embedded in Epon (3.61 M Glycidether 100, 1.83 M Methylnadicanhydride, 0.92 M Dodecenylsuccinic anhydride, 5.53 mM 2,4,6-Tris (dimethylaminomethyl)phenol. Ultrathin sections were sliced with an Ultramicrotome (Ultracut E; Reichert und Jung, Germany) and automatically stained with UranyLess EM Stain (Electron Microscopy Sciences) and 3% lead citrate using the contrasting system Leica EM AC20 (Leica, Wetzlar, Germany). The samples were examined with an JEOL − 1200 EXII transmission electron microscope (JEOL GmbH, Freising, Germany). Buffers were purchased from Serva Electrophoresis GmbH. Mitochondrial area and Feret diameter were analyzed identically for all samples using ImageJ.

### Seahorse

Metabolic activity was analyzed using an Agilent Seahorse 96XF device and respective kits. Cell mito stress test was performed as described in the manufacturer’s protocol (Kit 103015–100). Mitochondrial fuel dependency and capacity were determined according to manufacturer’s protocol (Kit 103270–100). Briefly, cells were pre-treated, medium was exchanged for seahorse medium. Compounds were present during the entire measurement. Respiratory parameters, fuel dependency and capacity were calculated using the Seahorse Wave Desktop Software and the Seahorse XF Cell Mito Stress Test Report Generator or the Seahorse XF Mito Fuel Flex Test Report Generator (Agilent Technologies).

### NADPH/NADP+ measurements

NADP+/NADPH levels were assessed using the NADP/NADPH-Glo™ Assay according to manufacturer’s protocol (Promega). Briefly, cells were treated as indicated (24 h). Media was replaced with PBS and basic lysis solution was added. Lysates were transferred to white walled 96-well plate and splitted for NADP+ and NADPH measurements. Respective solutions were added and NADP/NADPH-Glo™ Detection Reagent was added. After 60 min incubation at 25 °C luminescence was detected using an Orion II microplate luminometer (Berthold Detection Systems, Pforzheim, Germany).

### Proliferation

Proliferation was assessed with the CellTiter-Blue® Cell Viability Assay (Promega, Madison, WI, USA). 5,000 cells/well were seeded, basal metabolic activity was determined (24 h) and cells were treated as indicated for 72 h. CellTiter-Blue® Reagent was added for 4 h and the absorbance at 590 nm was measured in a Sunrise ELISA reader (Tecan, Maennerdorf, Austria) and is proportional to the cell number.

### Statistics

Experiments have been performed at least three times, unless stated otherwise. For analysis representative images out of three independent experiments are shown. Bars are the mean + SEM of three independent experiments. *P* values of p* < 0.05 (One-way ANOVA, Dunnett post test or student t-test) were considered significant.

## Results

### Impaired lysosomal function changes cellular lipid profile

**Fig. 1:**
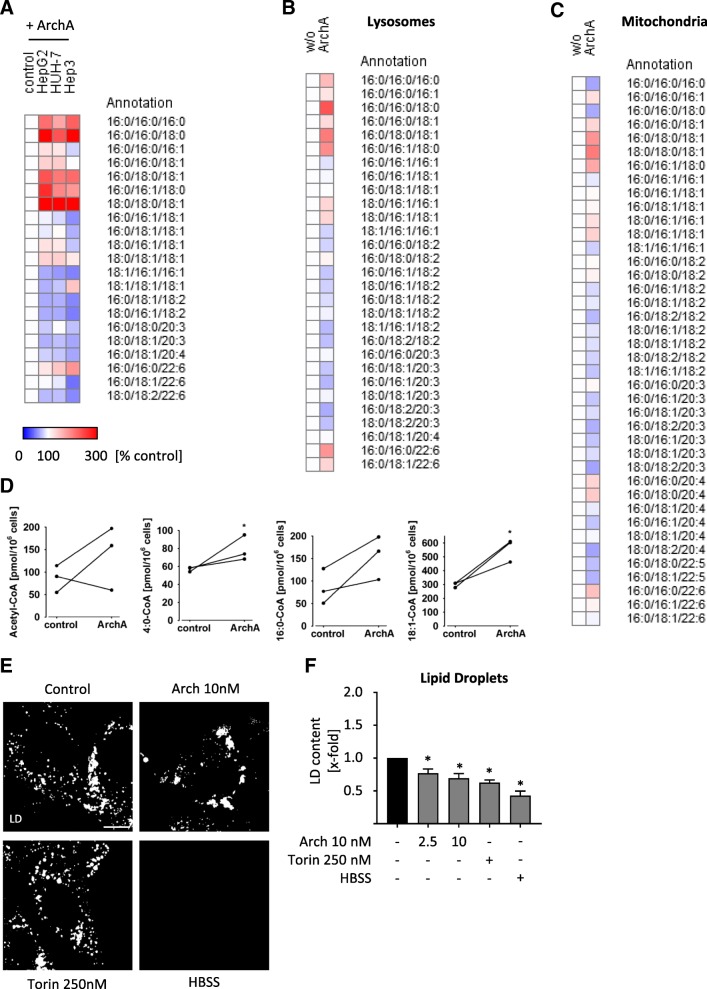
V-ATPase inhibition influences lipid profile. Cells were treated as indicated (24 h). Lipids from whole cells (HUH7, HepG2 and Hep3B) (**a**), lysosomes (HUH7) (**b**) or mitochondria (HUH7) (**c**) were isolated and TAG composition was analyzed by UPLC-MS/MS. Heatmaps display percentage increase (red) and decrease (blue) of respective TAG species compared to DMSO control. **d** Lipids from whole cells (HUH7) were isolated and cholesteryl ester composition was analyzed by mass spectrometry (student t-test). **e**, **f** Cells were loaded with Bodipy 493/503 to stain lipid droplets (LD). **e** LD size and localization was analyzed by confocal microscopy. Scale bar 10 μm. Representative images out of three independent experiments are shown. Bars are the mean + SEM of three independent experiments. **f** LD content was quantified by flow cytometry. p* < 0.05 (One-way ANOVA, Dunnett post test)

We recently showed, that lysosomal malfunction leads to alterations in cholesterol metabolism and subsequently to impaired proliferation of cancer cells [9]. To decipher the role of the lysosome in lipid regulation of cancer cells we disrupted lysosomal function by treatment with archazolid (Arch). Archazolid is a potent inhibitor of the lysosomal V-ATPase, which causes a drastic increase in luminal pH and thereby disrupts lysosomal function. Arch has shown promising anti-cancer activity in various studies [9, 10, 21–23]. We treated different hepatocellular carcinoma (HCC) cell lines with Arch for 24 h and subsequently analyzed composition of triacylglycerid species (TAG). We found that composition of TAG is strongly changed upon V-ATPase inhibition (Fig. 1a) shifting a lipid profile with an increased degree of saturation, while total TAG content is barely affected (Additional file 1: Figure S1A). The relative abundance of different lipid species in the HCC cell lines was comparable containing predominantly TAG with mono- and poly-unsaturated fatty acids (Additional file 1: Figure S1B-D). Furthermore, we were interested in the lipid composition of different organelles after Arch treatment. Hence, we isolated lysosomes and mitochondria of HUH7 cells after treatment and again analyzed TAG composition. In comparison to whole cells (Fig. 1a), TAG composition of lysosomes (Fig. 1b) was altered in the same manner, while palmitic acid containing TAGs were downregulated in mitochondria (Fig. 1c), total TAG content of isolated organelles did not change (Additional file 1: Figure S1E-F). Along the line, we also observed changes in Acyl-CoA levels after V-ATPase inhibition (Fig. 1d). Next, we investigated condition and content of lipid droplets (LD), the lipid storage organelles. In order to assess whether our observations are specific to V-ATPase inhibition or rather a general response to lysosomal stress, we included treatment with the mTOR inhibitor Torin 1 and starvation with HBSS, which have been shown to induce lysosomal stress and create a similar metabolic phenotype as compared to V-ATPase inhibition [24–26]. We observed that lysosomal stress in general leads to a change in LD size and distribution (Fig. 1e), as well as a decrease in overall LD content (Fig. 1f). Yet, localization of LD was varied between different stress conditions (Fig. 1E). Overall, we found that impairment of lysosomal function changes cellular lipid profile and subcellular localization of lipids.

### V-ATPase inhibition leads to alterations in lipid metabolism

**Fig. 2:**
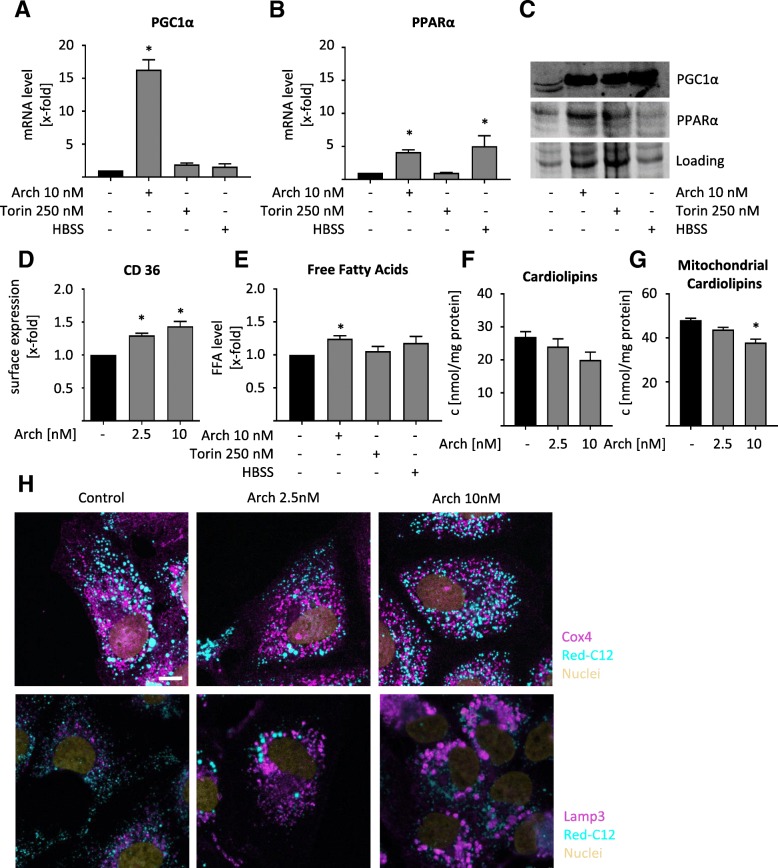
Lysosomal stress alters lipid metabolism. HUH7 cells were treated as indicated (24 h). Relative mRNA expression levels of PGC1α (**a**) and PPARα (**b**) were detected by qPCR. **c** Protein expression of PGC1α and PPARα was detected by WB. Total protein served as loading control. **d** CD36 expression was visualized by antibody staining and determined by flow cytometry. **e** Levels of free fatty acids were determined by a coupled enzyme assay, which results in a colorimetric product proportional to the fatty acids present (MAK044 Sigma Aldrich). Absorbance was quantified relative to DMSO control. **f**, **g** Cardiolipin content was analyzed using a fluorimetric detection kit (K944 Biovision) in whole cell lysate (**f**) and in isolated mitochondria (**g**). **h** Cells were labeled with Bodipy 558/568 Red C-12 (cyan). Cox4 and Lamp3 (magenta) were visualized by antibody staining and nuclei (yellow) by Hoechst 33342, respectively. Cells were analyzed by confocal microscopy. Scale bar 7.5 μm. Representative images out of three independent experiments are shown. Bars are the mean + SEM of three independent experiments. p* < 0.05 (One-way ANOVA, Dunnett post test)

Alterations in lipid composition might in principle arise from changes in synthesis, uptake or degradation processes, which we analyzed one after another. A crucial regulator of lipid metabolism is PGC1α. PGC1α is a master regulator of cellular energy metabolism, including mitochondrial beta oxidation, i.e. degradation of lipids to generate energy. Additionally, PGC1α is controlling lipid metabolism by transcriptional regulation of PPARα, which promotes uptake, utilization, and catabolism of fatty acids. Interestingly, 4:0 Co-A, an intermediate of beta-oxidation was significantly increased after Arch treatment (Fig. 1d). Quantitative real-time PCR (qPCR) measurements revealed that inhibition of V-ATPase tremendously increases PGC1α expression, while mTOR inhibition and starvation do not (Fig. 2a). Additionally, mRNA (Fig. 2b) and protein level (Fig. 2c) of PPARα is upregulated upon V-ATPase treatment. These data suggest that cells specifically upregulate catabolism of lipids upon treatment with Arch. Of note, other relevant downstream targets of PGC1α, namely NRF1, NRF2 and ERRα are not influenced in their expression upon induction of lysosomal stress (Additional file 2: Figure S2A-C). Furthermore, cells increase uptake of fatty acids as the surface expression of CD36, also called fatty acid translocase, is increased upon V-ATPase inhibition (Fig. 2d). Additionally, the level of free fatty acids, which are essential for energy generation in mitochondria are increased after Arch treatment (Fig. 2e). These findings strongly suggest that cells induce lipid degradation, plausibly to sustain energy generation upon V-ATPase inhibition. Free fatty acids can be converted to acetyl-CoA by mitochondria via ß-oxidation, feeding into the TCA cycle and fueling ATP synthesis via oxidative phosphorylation. For this, proper mitochondrial function is essential, in particular mitochondrial membrane composition is important, as the respiratory complexes are assembled there. As we observed specific alterations in TAG in mitochondria after treatment with Arch (Fig. 1c), we hypothesized that also cardiolipin content might be affected. Cardiolipins are a special lipid species representing essential mitochondrial inner-membrane lipids, essentially contributing to membrane curvature and therefore mitochondrial function [27]. We found that the content of cardiolipins is reduced in cells after V-ATPase inhibition (Fig. 2 F-G), indicating mitochondrial malfunction. Cells are furthermore able to facilitate lipids by degradation in lysosomes, a process called lipophagy. In order to determine lipid localization, we analyzed colocalization of a labelled C-12 lipid with lysosomes and mitochondria (Fig. 2h, Additional file 2: Figure S2D-E), yet no significant overlay could be detected, indicating that lipophagy is not predominant. However, confocal images indicate a shift of mitochondrial structure from long networks to short, round-shaped mitochondria (Cox4) (Fig. 2h) upon V-ATPase inhibition, indicating mitochondrial fission.

### Lysosomal mediated structural changes in mitochondria

**Fig. 3:**
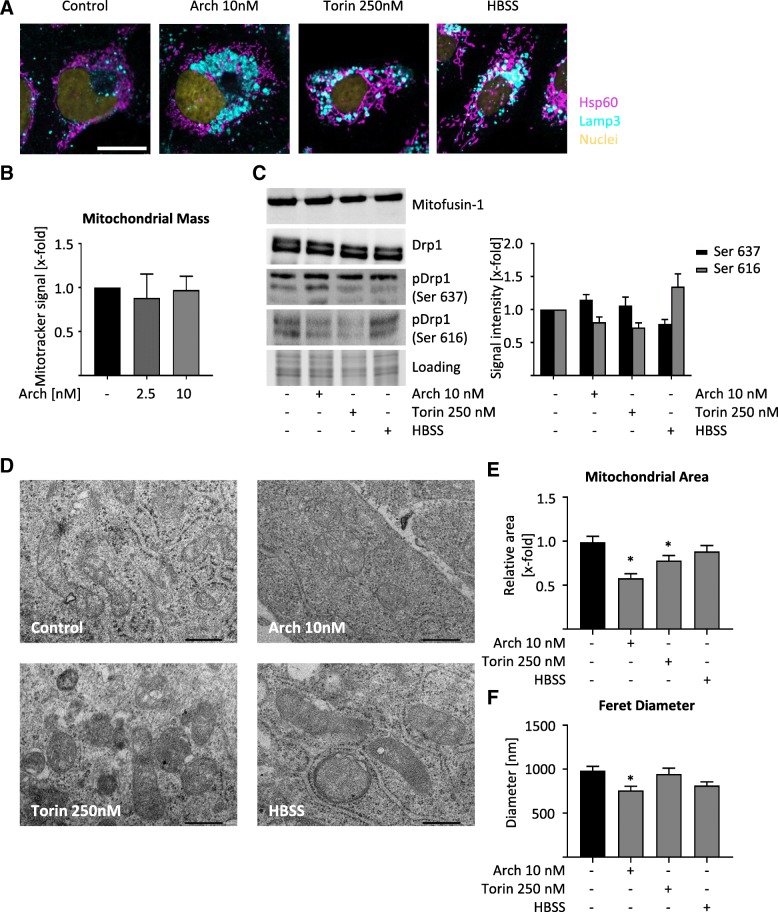
Mitochondrial structure is changed upon V-ATPase inhibition. HUH7 cells were treated as indicated (24 h). **a**, **c**, **d** Representative images out of three independent experiments are shown. **a** Cells were fixed and stained for the mitochondrial marker Hsp60 (magenta), lysosomal marker Lamp3 (cyan) and nuclei (yellow) and analyzed by confocal microscopy. Scale bar 25 μm. **b** Mitochondria were stained with MitoTracker™ Green FM and mitochondrial mass was assessed by flow cytometry immediately. **c** Protein level was detected by WB. Total protein served as loading control. Quantification of Drp1 phosphorylation (bar graph). **d** Mitochondrial morphology was analyzed by TEM. Scale bar 500 nm. **e** Relative change in mitochondrial area was assessed using ImageJ. At least fifty mitochondria from TEM images (**d**) have been analyzed. **f** Feret diameter of at least fifty mitochondria from TEM images (**d**) was calculated using ImageJ. Bars are the mean + SEM of three independent experiments. p* < 0.05 (One-way ANOVA, Dunnett post test)

Mitochondrial fission is implicated in the cellular stress response and apoptosis and it usually precedes mitochondrial degradation by lysosomes, i.e. mitophagy [16, 28]. A co-staining of lysosomes and mitochondria showed no overlay after V-ATPase or mTOR inhibition, but after starvation, displaying an indication for mitophagy (Fig. 3a). Flow cytometry furthermore revealed that the mitochondrial mass is not changed after V-ATPase inhibition (Fig. 3b), indicating that no significant degradation of mitochondria is induced. Western Blot analysis of Mitofusin-1 and Drp1 confirmed an increased phosphorylation of Drp1, confirming the fission phenotype, since phosphorylated Drp1 mediates the final step of fission, the separation to two daughter mitochondria (Fig. 3d, Additional file 3: Figure S3A). Detailed structural analysis of mitochondria in electron microscopy displayed rather elongated mitochondria in control cells and smaller, round-shaped mitochondria in Arch treated cells (Fig. 3d, Additional file 3: Figure S3B). This is also reflected after analyzing mitochondrial area in EM images (Fig. 3e) and Feret diameter (Fig. 3f). Hence, we hypothesize that cancer cells induce mitochondrial fission after V-ATPase inhibition in order to sustain sufficient energy production.

### Lysosomal stress disrupts mitochondrial function

**Fig. 4:**
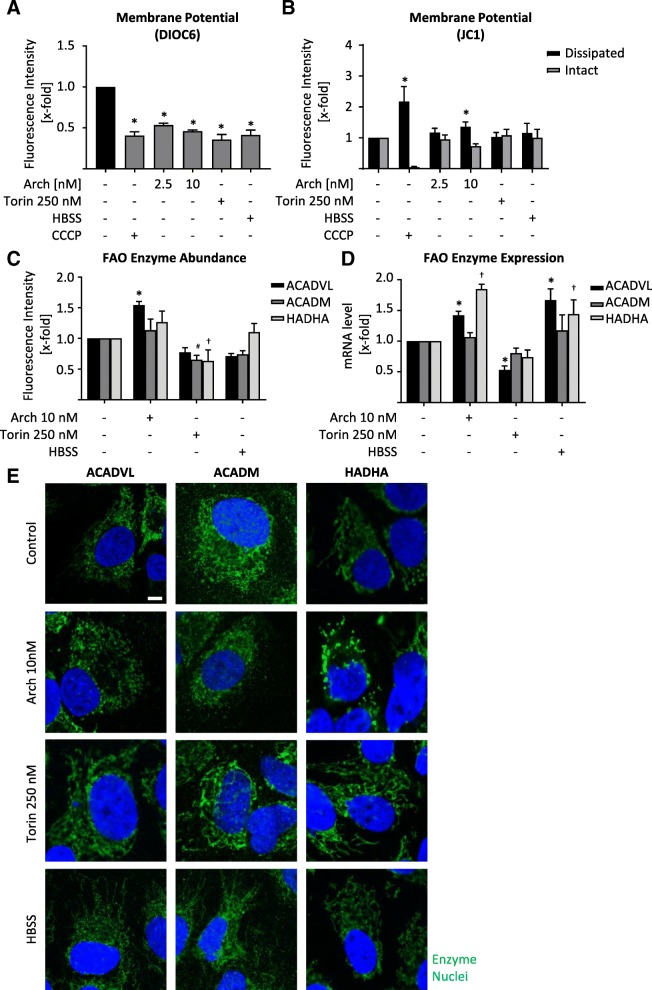
Alterations in mitochondrial function due to lysosomal stress. HUH7 cells were treated as indicated (24 h). DIOC6 (**a**) or JC1 (**b**) fluorescence were quantified by flow cytometry. **c** Enzyme abundance was quantified by flow cytometry. **d** Relative mRNA expression levels of ACADVL*, ACADM^#^ and HADHA^†^ were assessed by qPCR. **a-d** Bars are the SEM of three independent experiments. p*^#†^ < 0.05 (One-way ANOVA, Dunnett post test) (**e**) Cells were fixed and stained for ACADVL, ACADM and HADHA, respectively, (green) and nuclei (blue) and analyzed by confocal microscopy. Scale bar 7.5 μm. Representative images out of three independent experiments are shown

**Fig. 5:**
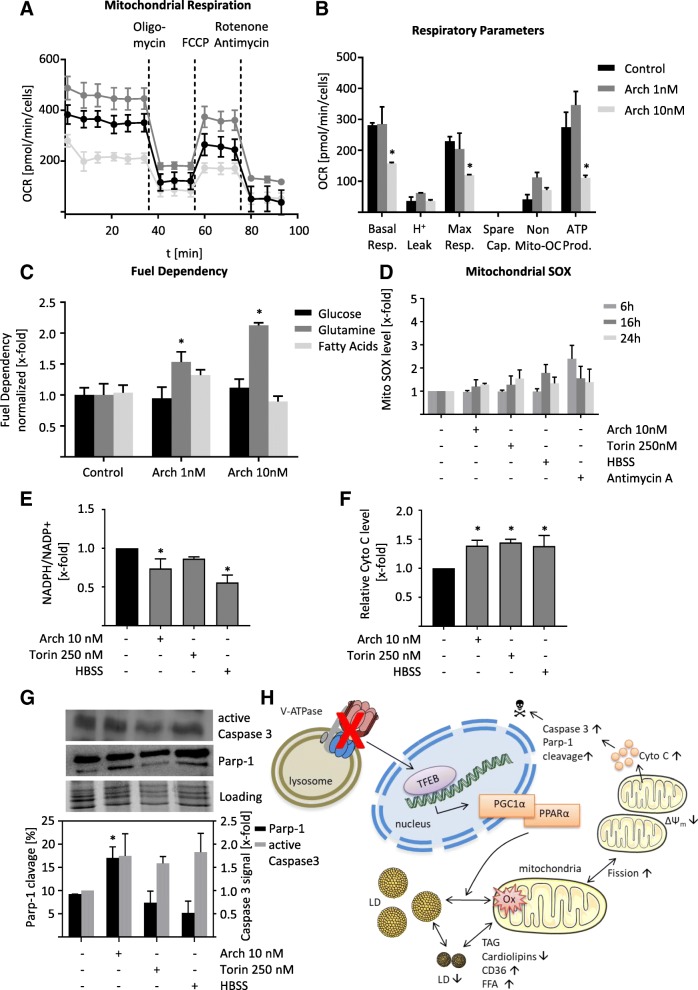
V-ATPase inhibition alters cellular metabolism. HUH7 cells were treated as indicated (24 h). **a** Cells were exposed sequentially to oligomycin, FCCP and rotenone/antimycin. Vertical lines indicate time of addition of mitochondrial inhibitors. Oxygen consumption rate (OCR) was measured over time using a Seahorse XFe96 Analyzer. Cell mito stress test was performed according to manufacturer’s protocol. **b** Respiratory parameters were calculated from OCR data (**a**) according to mitochondrial stress test protocol (User Guide Kit 103015–100 Agilent). **c** Mitochondrial fuel flex test was performed according to manufacturer’s protocol (User Manual Kit 103270–100 Agilent). Fuel dependency was calculated as described in the manual. **d** Cells were loaded with MitoSOX™ and mitochondrial superoxide (SOX) was quantified by flow cytometry. **e** NADPH and NADP+ levels were assessed using NADP/NADPH-Glo™ luminescence-based assay as described by the manufacturer (G9081 Promega). NADPH to NADP+ ratio was calculated and normalized to DMSO control. **f** Cytosolic cytochrome C was detected by flow cytometry. Bars are the mean + SEM of three independent experiments. p* < 0.05 (One-way ANOVA, Dunnett post test) (**g**) Protein levels of active Caspase 3 and Parp-1 cleavage have been determined and quantified by Western Blot. **h** Cartoon of mode of action. V-ATPase inhibition leads to transcriptional regulation of PGC1α and PPAR α. Lipid droplets (LD) are changed in size and localization, leading to cardiolipin depletion, fission and impairment of mitochondrial function. This results in induction of mitochondria-driven apoptosis

As we observed mitochondrial fission after V-ATPase inhibition, we checked whether mitochondria are still functional. Flow cytometric analysis of cells stained with DIOC6, a dye that is selectively localized to intact mitochondria, revealed that mitochondrial membrane potential is disrupted indicating a loss of mitochondrial function (Fig. 4a). A staining with JC1 confirmed this finding, by displaying a high amount of mitochondria with dissipated membrane potential (Fig. 4b). The effect of Arch on mitochondrial membrane potential was almost as prominent as carbonyl cyanide m-chlorophenyl hydrazine (CCCP), a known uncoupling agent. To further characterize the state of mitochondrial function, we analyzed transcription, protein abundance and localization of important enzymes in fatty acid beta oxidation, namely ACADVL, ACADM and HADHA. These enzymes catalyze the first steps of fatty acid beta oxidation and differ in affinity towards fatty acids with different chain lengths. While ACADVL is specific for very long chain fatty acids, ACADM is specific for medium chain fatty acids and HADHA catalyzes three out of four steps of beta-oxidation of long chain fatty acids. After V-ATPase inhibition protein and expression level of ACADVL are increased. mTOR inhibition rather led to a decrease in enzyme abundance, while starvation showed no consistent alterations (Fig. 4c, d, Additional file 4: Figure S4A). Confocal imaging of enzyme localization confirmed the Arch induced fission phenotype as observed before (Fig. 4e). These data indicate a reduction in mitochondrial function. To prove this impairment, we performed seahorse measurements facilitating a mitochondrial stress test. After pre-treatment of the cells with the respective compounds, oxygen consumption rate (OCR) was measured over time and inhibitors of mitochondrial function (oligomycin, FCCP and Rotenone+Antimycin) were added sequentially (Fig. 5a) in order to calculate respiratory parameters of the cells (Fig. 5b). After treatment with Arch the basal respiration, as well as maximum respiratory capacity and ATP production are strongly decreased compared to control cells (Fig. 5a, b), confirming an impairment in mitochondrial function. The reduction in ATP levels could also be confirmed in a CellTiter-Glo® assay (Additional file 4: Figure S4B). Additionally, we used the mitochondria fuel flex test to investigate which source the cells mainly use for beta oxidation. This test blocks different fuels from being oxidized in the mitochondria by using the specific inhibitors BPTES, which inhibits conversion of glutamine to glutamate, UK50699, which inhibits mitochondrial pyruvate carrier, therefore blocking glucose as energy source and etomoxir, which inhibits palmitate uptake into mitochondria and therefore blocks fatty acid oxidation. We found that upon V-ATPase inhibition cells become more dependent on glutamine as energy source (Fig. 5c), while the capacity to use each single energy source remains unchanged (Additional file 4: Figure S4C). Furthermore, we analyzed mitochondrial superoxide (SOX) levels, yet found only a slight increase after 24 h (Fig. 5d). Also, on a cellular level, ROS was not increased after 24 h (Additional file 4: Figure S4D). However, NADPH/NADP+ ratio was significantly decreased after 24 h hinting to a deregulation in cellular ROS (Fig. 5e), apparent at later timepoints. Deregulation in mitochondrial function, disruption of mitochondrial membrane potential and ROS generation are key players in induction of the mitochondrial apoptosis pathway. Indeed, we found an increase in cytosolic cytochrome C (Fig. 5f) as well as an increase in caspase 3 activation and Parp-1 cleavage after V-ATPase inhibition (Fig. 5g). Taken together, our data clearly show that lysosomal stress leads to alterations in LD and cardiolipin content subsequently an impairment of mitochondrial function, a reduction in energy generation and an induction of mitochondria-driven apoptosis (Fig. 5h).

### V-ATPase inhibition causes metabolic shift

**Fig. 6:**
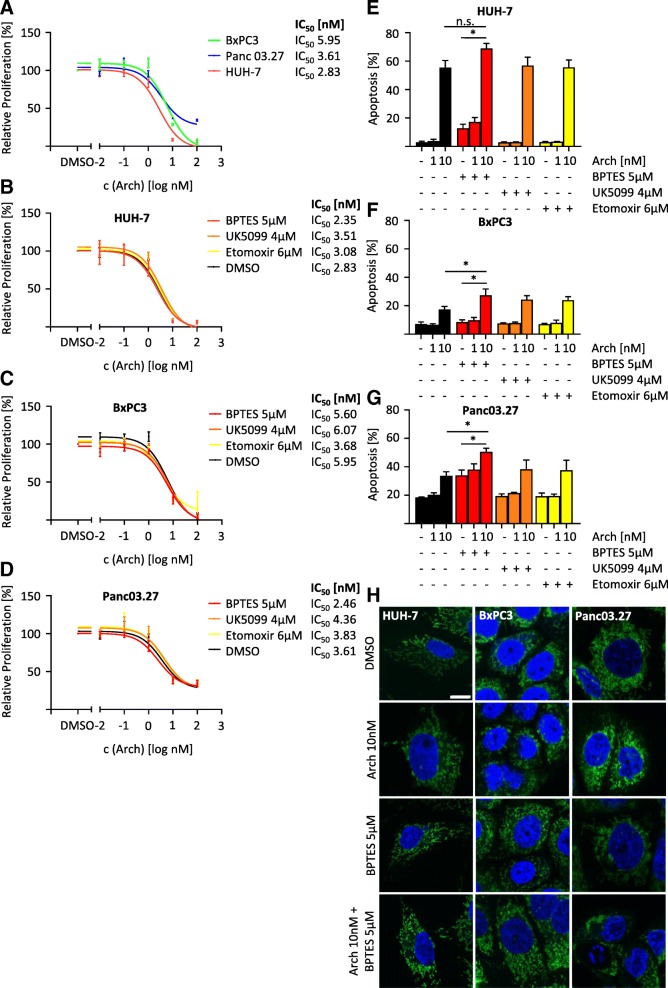
Glutamine deprivation sensitizes cancer cells towards V-ATPase inhibition. **a** Dose response curves for Arch treatment were determined after 72 h. Dose response curves for Arch in the presence or absence (DMSO) of a constant concentration of BPTES (5 μM), UK5099 (4 μM) or etomoxir (6 μM) of HUH7 (**b**), BxPC3 (**c**) and Panc03.27 (**d**) were determined after 72 h (**a**-**d**) Cell viability was analyzed by CellTiter Blue assay. IC_50_ values were calculated by non-linear curve-fit using GraphPad Prism. HUH7 (**e**), BxPC3 (**f**) and Panc 03.27 (**g**) were treated as indicated for 48 h. Apoptotic cells were determined by Nicoletti assay. **h** HUH7, BxPC3 and Panc03.27 were treated as indicated for 24 h, stained for mitochondria and nuclei and analyzed by confocal microscopy. Scale bar 10 μm. Values are the mean + SEM of three independent experiments. p* < 0.05 (One-way ANOVA, Tukey post test)

Since we discovered an increased glutamine dependency after V-ATPase inhibition (Fig. 5c), we were interested whether this can be exploited for novel therapeutic strategies. We used three different cell lines with varying Ras mutational status, as Ras mutations have been connected to mitochondrial function in the past [29, 30]. First, we determined sensitivity to Arch in a proliferation assay, which showed that Ras wild-type cells (BxPC3 IC_50_ 5.95 μM) are less sensitive to the treatment than cells carrying Ras pathway mutations (Panc 03.27 IC_50_ 3.61 μM, HUH7 IC_50_ 2.83 μM) (Fig. 6a). We subsequently determined dose-response curves on proliferation inhibition in the presence or absence of the same metabolic inhibitors as used in the seahorse fuel flex test (Fig. 6b-d). This revealed that presence of BPTES, a glutaminase inhibitor, slightly decreased the IC_50_ values of Arch in all cell lines. Furthermore, inhibition of the glutamine pathway increased the capacity of Arch to induce apoptosis in all tested cell lines, with the greatest effect in Panc 03.27 cells (Fig. 6e-g). The potential to induce apoptosis is linked to the induction of a fission phenotype, as fission correlated with the ability to induce apoptosis (Fig. 6h).

## Discussion

In this study, we unravel a novel role for lipid metabolism in cancer cells, linking lysosomal disturbance to an induction of mitochondrial apoptosis. We show that induction of lysosomal stress, especially by inhibition of the V-ATPase, leads to disturbance in cancer cell lipid content, which ultimately disrupts mitochondrial function.

The view of the lysosome has been changing dramatically in the recent past. Having simply been viewed as cellular trash can, the lysosome turns out to be a central regulator of cellular function and has been shown to be of particular importance in energy regulation [1]. Settembre and colleagues discovered that lysosomes are essential for maintaining cellular homeostasis, by sensing lysosomal nutrient status and inducing adaptations to starvation via a mechanism facilitating mTORC1 and transcription factor EB (TFEB). Upon lysosomal stress, TFEB translocates to the nucleus and induces lysosomal biogenesis [25]. Furthermore, the same group also discovered a role of TFEB in lipid regulation. They show that starvation induces TFEB translocation, which induces global transcription control of lipid catabolism and lipophagy by induction of PGC1α and PPARα transcription [24]. Our data are in accordance with their findings, as we could also detect an induction of PGC1α and PPARα. Yet, we suggest that this is not entirely mediated by TFEB translocation or translocation of TFE3 or MITF, two transcription factors of the same family that have also been connected to lysosome-to-nucleus signaling [31–33]. In our experiments, we could only detect a slight upregulation of nuclear translocation of TFEB, but not TFE3 or MITF, in HUH7 cells (Additional file 5: Figure S5A-C) using specific antibodies. However, we could confirm their data using constitutively active TFEB in HEK-293 cells (Additional file 5: Figure S5D). We hypothesize that upregulation of TFEB, PGC1α and PPARα as well as increasing lipid uptake via CD36 as discovered in our study is an escape mechanism, by which the cancer cells try to compensate for defective mitochondria and loss of energy induced by V-ATPase inhibition.

Mitochondrial function is crucially dependent on proper mitochondrial structure. This is especially true for the mitochondrial membrane. Mitochondria are double membrane bound organelles consisting of an inner and an outer membrane. The inner membrane displays a characteristic curvature that forms cristae, which are pivotal for mitochondrial respiration [16, 27]. A highly abundant lipid species of the mitochondrial inner membrane are cardiolipins, which are crucial for energy generation by oxidative phosphorylation [34]. Furthermore, mitochondria play a central role in regulating apoptosis. In their inter-membrane space various proteins are stored, which can be released due to pro-apoptotic stimuli and activate caspase dependent cell death. Following pro-apoptotic stimuli, Bcl-2 family proteins Bak and Bax translocate to mitochondria and form a pore through which cytochrome C is released from the inter-membrane space into the cytosol to activate caspases [35]. In our study, we observed perturbations in lipid composition, in a manner that the level of saturated fatty acids increases, while the level of desaturated fatty acids decreases. Furthermore, we found a decrease in mitochondrial cardiolipins, which may lead to the observed mitochondrial dysfunction. While we were not able to detect significant translocation of Bak or Bax to the mitochondria (Additional file 4: Figure S4E), we observed cytochrome C release, caspase activation and Parp cleavage, i.e. induction of apoptosis. Induction of apoptosis caused by saturated fatty acids has been observed also by others [36–38]. It was even reported that there is a differential role of saturated and unsaturated fatty acids, whereas unsaturated fatty acids influence autophagy but do not promote apoptosis, saturated fatty acids suppress autophagy and induce apoptosis [39]. However, other studies found that unsaturated lipids can induce apoptotic cell death, especially when peroxidized [40, 41]. We conclude that the observed changes in lipid composition caused by disruption of lysosomal function lead to a change in mitochondrial membrane composition, which on the one hand impairs mitochondrial function and on the other, triggers release of cytochrome C to the cytosol and subsequently induces apoptosis. This concept of inter-organelle cross talk between lysosomes and mitochondria is just emerging [15]. There is evidence that there exist direct contact sites between lysosomes and mitochondria in yeast, which facilitate the exchange of ions and phospholipids between the organelles [42, 43]. Yet, direct contact sites have only recently been discovered in skeletal muscle [44]. Interestingly, it has also been shown that lysosomal targeted formulations of 5-FU containing nanogels localize to the lysosomes and trigger mitochondria-driven apoptosis [45]. This phenomenon was induced by release of cathepsin B from lysosomes, which activated caspase 9, yet the arch induced mechanism cannot be identical, as our group could previously show that the level of active cathepsin B is reduced after treatment [46]. Whether there exist direct contact sites in cancer cells or how these organelles exactly interact remains to be elucidated, however our data suggest that the two organelles strongly interact at least on a functional level. Additionally this study sheds more light on the mechanism by which V-ATPase inhibition leads to mitochondria-driven apoptosis, which our group already investigated in the past [10].

Interestingly, oncogenic Ras signaling has been connected to mitochondrial function. Ras is a well-established oncogene, which is hyperactive in a variety of cancers [47, 48]. Ras exists in three isoforms, namely K-Ras, H-Ras and N-Ras, all of which display oncogenic potential. Additionally also Ras pathway mutations, downstream of Ras itself have been linked to cancer progression [49]. The exact mechanisms of Ras-induced malignant transformation are currently still investigated. Hu et al. could show that oncogenic K-Ras is associated with mitochondria where it changes the metabolic phenotype of the cells promoting Warburg effect [50] and drives tumor development [51]. Serasinghe and colleagues provide data supporting the findings of Hu et al. They show that an oncogenic Ras mutation is closely linked to changes in mitochondria. In their study they found that mitochondrial fission by Drp1 is essential for Ras malignant transformation and that also constitutively active MAPK signaling downstream of Ras induces mitochondrial changes in a Drp1 dependent manner [29]. As our previous work showed that inhibition of the V-ATPase is able to inhibit Ras pathway activation [9] and our current work connects lysosomes and mitochondria we assumed a possibility to address Ras mutated cells by manipulation of lysosomal function. Indeed, we found that K-Ras mutated Panc 03.27 cells and HUH7, which also show constitutive Ras pathway activation, are more sensitive to V-ATPase inhibition than the Ras wild-type BxPC3. These findings provide a first hint that Ras pathway mutations might sensitize cells for treatment with V-ATPase inhibitors, a connection that certainly should be addressed with future research.

## Conclusion

The present work provides a novel role for cellular lipid metabolism in the organelle cross-talk between lysosomes and mitochondria. Induction of lysosomal stress, especially by inhibiting the V-ATPase, leads to a reduction in mitochondrial cardiolipin content, induces fission, disrupts mitochondrial function and induces mitochondria-driven apoptosis. The study enhances our understanding of the interaction between lysosomes and mitochondria as well as the mechanism by which V-ATPase inhibition induces apoptosis. This increase in knowledge will help to develop new anti-cancer therapeutic strategies targeting the lysosome-mitochondria axis.

## Additional files

Additional file 1: **Figure S1.** (A) Cells were treated as indicated for 24 h. Lipids from whole cells (HUH7, HepG2 and Hep3B) were isolated. Total amounts of TAG were determined by UPLC-MS/MS and normalized to cell numbers. Bars are the mean+SEM of three independent experiments. p* < 0.05 (paired student t-test). Cells were treated with vehicle (DMSO) as indicated (24 h). Lipids from whole cells lysates of HUH7 (B), HepG2 (C), and Hep3B (D) were isolated, and the cellular proportion of TAG species was analyzed by UPLC-MS/MS. Lipids from isolated lysosomes (E) or mitochondria (F) were isolated. Total amounts of TAG were determined by UPLC-MS/MS and normalized to cell numbers. Bars are the mean+SEM of three independent experiments. p* < 0.05 (paired student t-test). Lipids from isolated lysosomes (G) and mitochondria (H) of HUH7 were isolated, and the cellular proportion of TAG species was analyzed by UPLC-MS/MS. Data are given as percentage of all TAG species detected (100%). Bars are the mean+SEM of three independent experiments. (PDF 196 kb)

Additional file 2: **Figure S2.** (A-C) HUH-7 cells were treated as indicated. Relative mRNA expression levels of NRF1 (A), NRF2 (B) and ERRα (C) were detected by qPCR. (D, E) Cells were labeled with Bodipy 558/568 Red C-12 (cyan) Bodipy 493/503 (magenta) and lysotracker (D) (yellow) or mitotracker (E) (yellow), repectively. Scale bar 25 μm. Representative images out of three independent experiments are shown. (PDF 441 kb)

Additional file 3: **Figure S3.** HUH-7 cells were treated as indicated. Representative images out of three independent experiments are shown. (A) Protein level was detected by WB. Total protein served as loading control. Quantification of Drp1 phosphorylation (bar graphs). (B) Confocal live cell imaging was performed staining for mitochondria (green), lysosomes (red) and nuclei (blue). Scale bar 25 μm. (PDF 574 kb)

Additional file 4: **Figure S4.** HUH-7 cells were treated as indicated. Representative images out of three independent experiments are shown. (A) Protein expression (24 h) of ACADVL, ACADM and HADHA was detected by WB. Total protein served as loading control. (B) Relative ATP levels were assessed by CellTiter-Glo® assay according to manufacturer’s protocol after 24 h treatment as indicated. (C) Mitochondrial Fuel Flex Test was performed according to manufacturer’s protocol (User Manual Kit 103270-100 Agilent) and capacity was calculated as described in the manual. (D) Cells were loaded with the redox sensitive dye Carboxy-H_2_DCFDA (DCF) and analyzed by flow cytometry. Quantification of DCF fluorescence intensity normalized to DMSO control. Bars are the SEM of three independent experiments. (E) Protein expression (24 h) of Bax and Bak was detected by WB on isolated mitochondria. Total protein served as loading control. (PDF 472 kb)

Additional file 5: **Figure S5.** HUH-7 cells were treated as indicated. Representative images out of three independent experiments are shown. (A) Cells were fixed and stained for TFE3, MITF or TFEB, respectively (green) and nuclei (blue) and analyzed by confocal microscopy. Scale bar 10 μm. (B) Cell lysates were fractioned in nuclei and cytosolic fractions and was protein expression of TFE3, MITF and TFEB, was detected by WB. Total protein served as loading control. Nuclear levels of transcription factors were quantified (bar graphs) (C) Relative mRNA expression levels of TFEB, TFE3 and MITF were detected by qPCR. (D) Wilde-type TFEB and a consitutively active TFEB mutant (TFEB) tagged with GFP (green) were overexpressed in HEK 293 cells. Cells were fixed, co-stained for nuclei and analyzed by confocal microscopy. Scale bar 7.5 μm. (PDF 673 kb)

## Data Availability

All data generated or analyzed during this study are included in this published article and its supplementary information files.

## Abbreviations

EM: Electron microscopy
HBSS: Hank’s balanced salt solution
HCC: hepatocellular carcinoma LD lipid droplet
MAPK: Mitogen-activated protein kinase
ROS: Reactive oxygen species
TAG: Triacylglcerid
V-ATPase: Vacuolar H^+^-ATPase
WB: Western Blot
