# Supplementary figures and images for: Connecting lysosomes and mitochondria – a novel role for lipid metabolism in cancer cell death
Source: Cell Commun Signal. 2019 Jul 29;17:87. doi: 10.1186/s12964-019-0399-2 (PMC6664539; doi:10.1186/s12964-019-0399-2)

**A**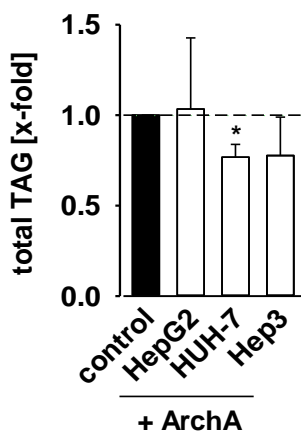**B**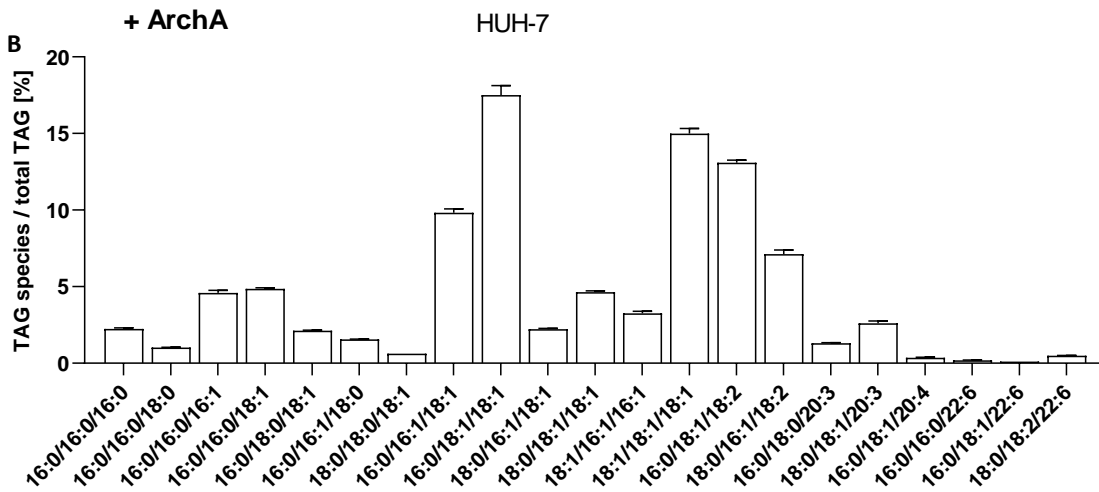**C**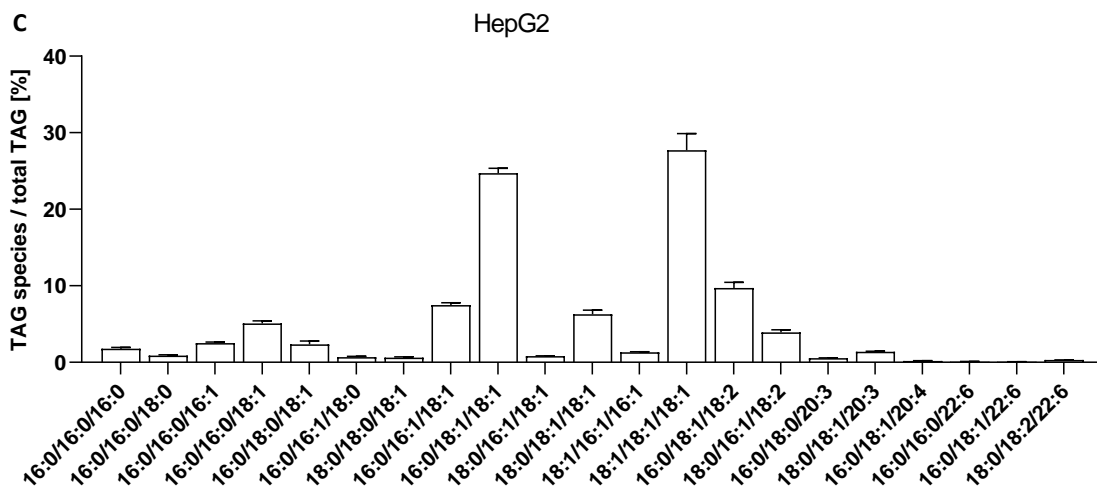**D**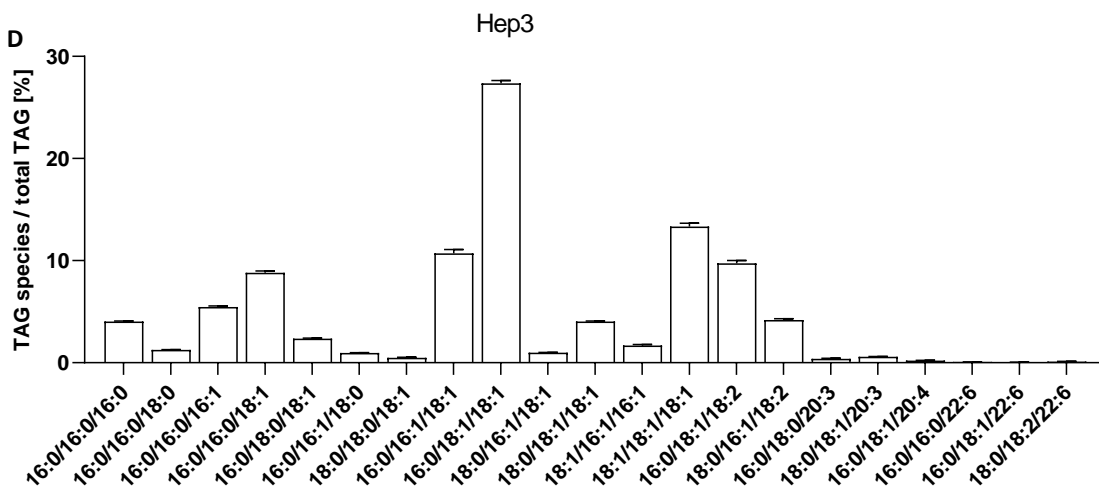

Supplement: Supplementary file 1 — Figure S1. (A) Cells were treated as indicated for 24 h. Lipids from whole cells (HUH7, HepG2 and Hep3B) were isolated. Total amounts of TAG were determined by UPLC-MS/MS and normalized to cell numbers. Bars are the mean+SEM of three independent experiments. p* < 0.05 (paired student t-test). Cells were treated with vehicle (DMSO) as indicated (24 h). Lipids from whole cells lysates of HUH7 (B), HepG2 (C), and Hep3B (D) were isolated, and the cellular proportion of TAG species was analyzed by UPLC-MS/MS. Lipids from isolated lysosomes (E) or mitochondria (F) were isolated. Total amounts of TAG were determined by UPLC-MS/MS and normalized to cell numbers. Bars are the mean+SEM of three independent experiments. p* < 0.05 (paired student t-test). Lipids from isolated lysosomes (G) and mitochondria (H) of HUH7 were isolated, and the cellular proportion of TAG species was analyzed by UPLC-MS/MS. Data are given as percentage of all TAG species detected (100%). Bars are the mean+SEM of three independent experiments. (PDF 196 kb) [file 12964_2019_399_MOESM1_ESM.pdf]

**A**

**NRF1**

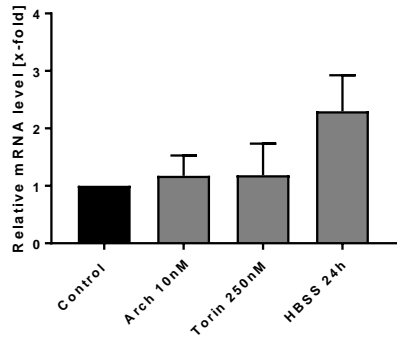

**B**

**NFR2**

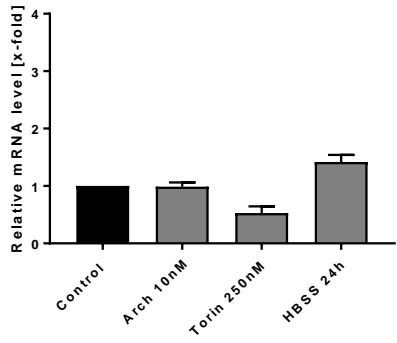

**C**

**ERRa**

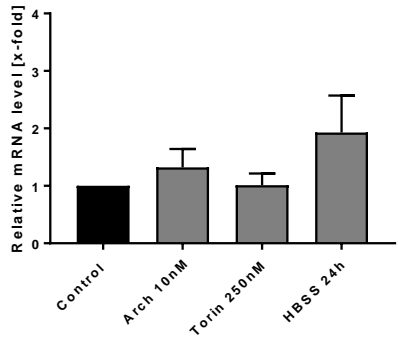

**D** Control Arch 10nM Torin 250nM HBSS

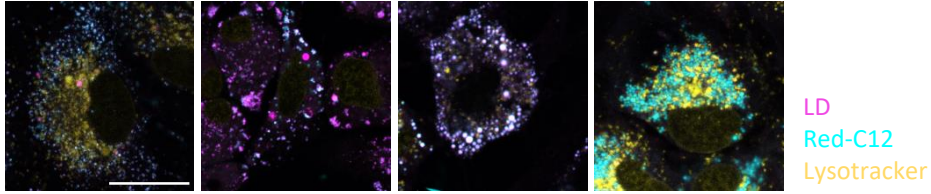

**E** Control Arch 10nM Torin 250nM HBSS

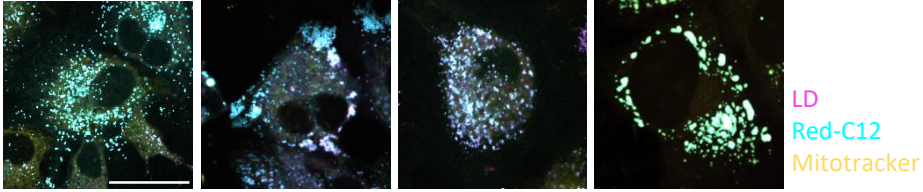

Supplement: Supplementary file 2 — Figure S2. (A-C) HUH-7 cells were treated as indicated. Relative mRNA expression levels of NRF1 (A), NRF2 (B) and ERRα (C) were detected by qPCR. (D, E) Cells were labeled with Bodipy 558/568 Red C-12 (cyan) Bodipy 493/503 (magenta) and lysotracker (D) (yellow) or mitotracker (E) (yellow), repectively. Scale bar 25 μm. Representative images out of three independent experiments are shown. (PDF 441 kb) [file 12964_2019_399_MOESM2_ESM.pdf]

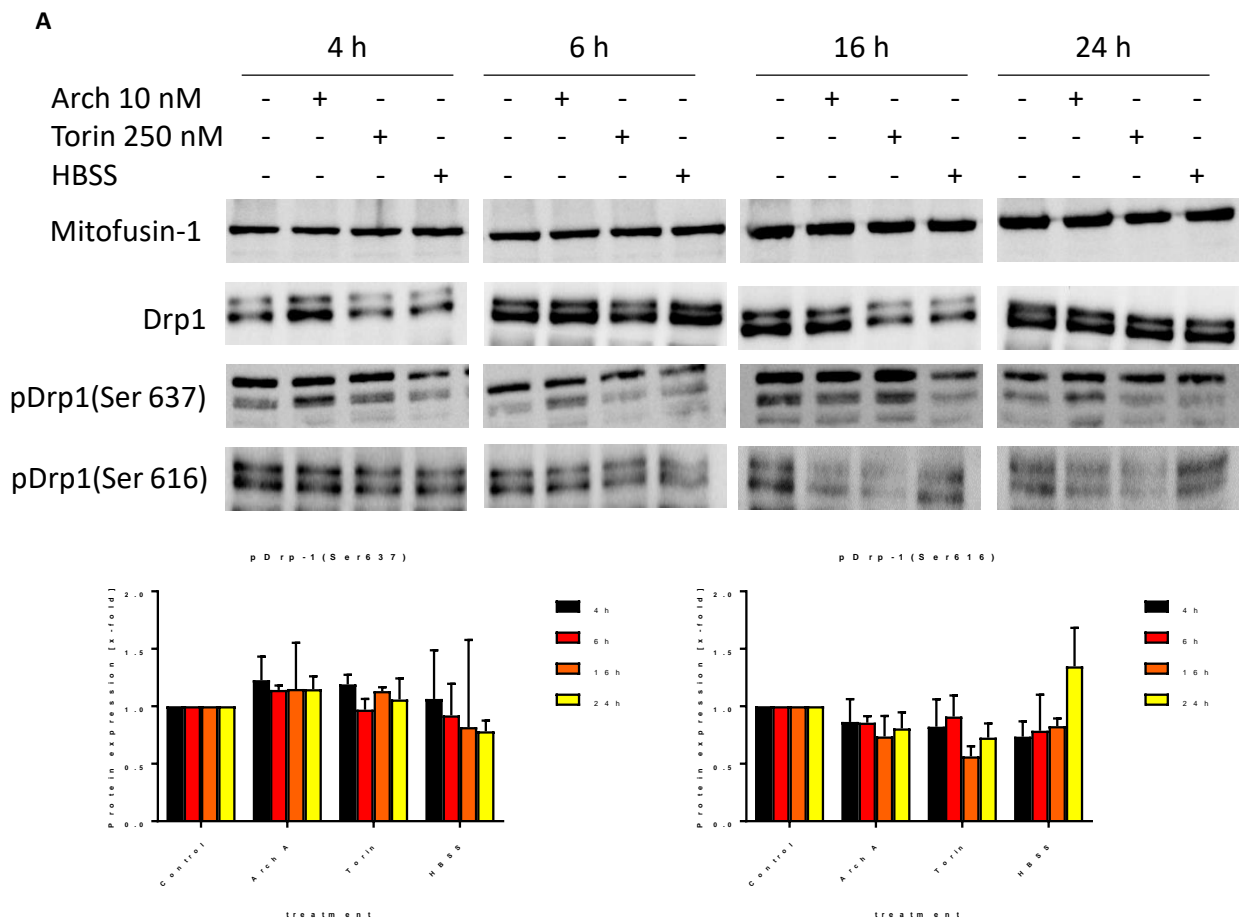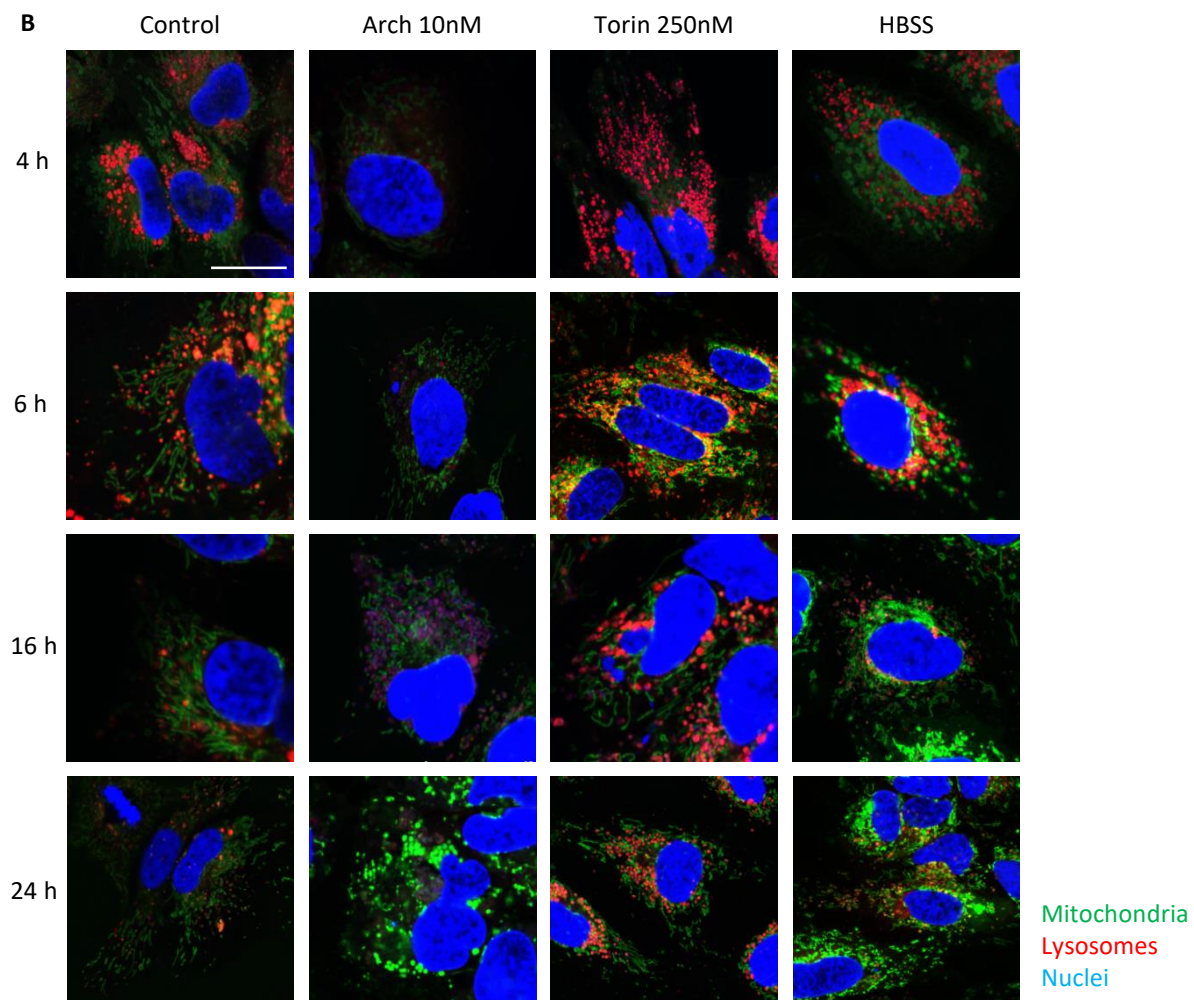

Supplement: Supplementary file 3 — Figure S3. HUH-7 cells were treated as indicated. Representative images out of three independent experiments are shown. (A) Protein level was detected by WB. Total protein served as loading control. Quantification of Drp1 phosphorylation (bar graphs). (B) Confocal live cell imaging was performed staining for mitochondria (green), lysosomes (red) and nuclei (blue). Scale bar 25 μm. (PDF 574 kb) [file 12964_2019_399_MOESM3_ESM.pdf]

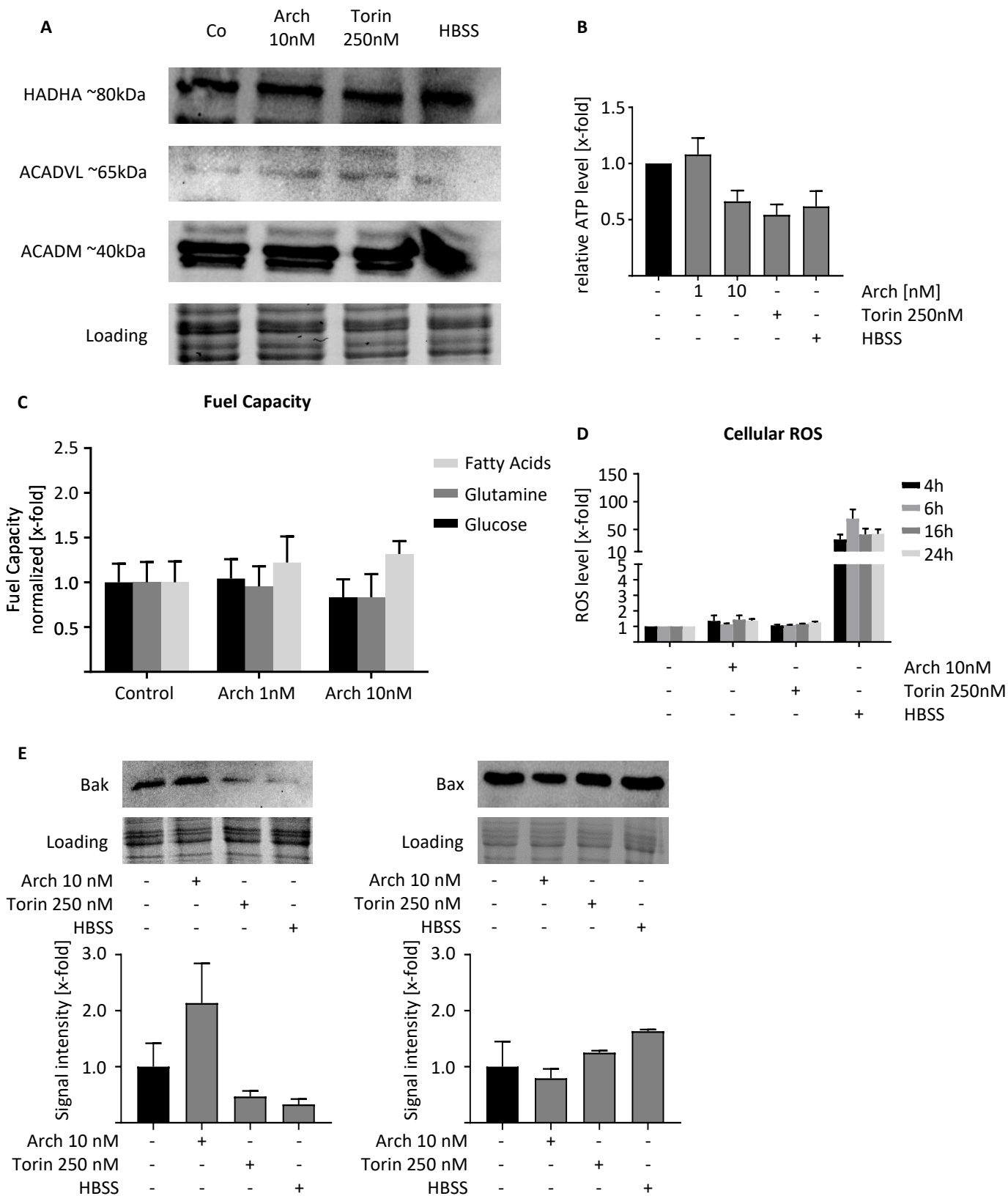

Supplement: Supplementary file 4 — Figure S4. HUH-7 cells were treated as indicated. Representative images out of three independent experiments are shown. (A) Protein expression (24 h) of ACADVL, ACADM and HADHA was detected by WB. Total protein served as loading control. (B) Relative ATP levels were assessed by CellTiter-Glo® assay according to manufacturer’s protocol after 24 h treatment as indicated. (C) Mitochondrial Fuel Flex Test was performed according to manufacturer’s protocol (User Manual Kit 103270-100 Agilent) and capacity was calculated as described in the manual. (D) Cells were loaded with the redox sensitive dye Carboxy-H2DCFDA (DCF) and analyzed by flow cytometry. Quantification of DCF fluorescence intensity normalized to DMSO control. Bars are the SEM of three independent experiments. (E) Protein expression (24 h) of Bax and Bak was detected by WB on isolated mitochondria. Total protein served as loading control. (PDF 472 kb) [file 12964_2019_399_MOESM4_ESM.pdf]

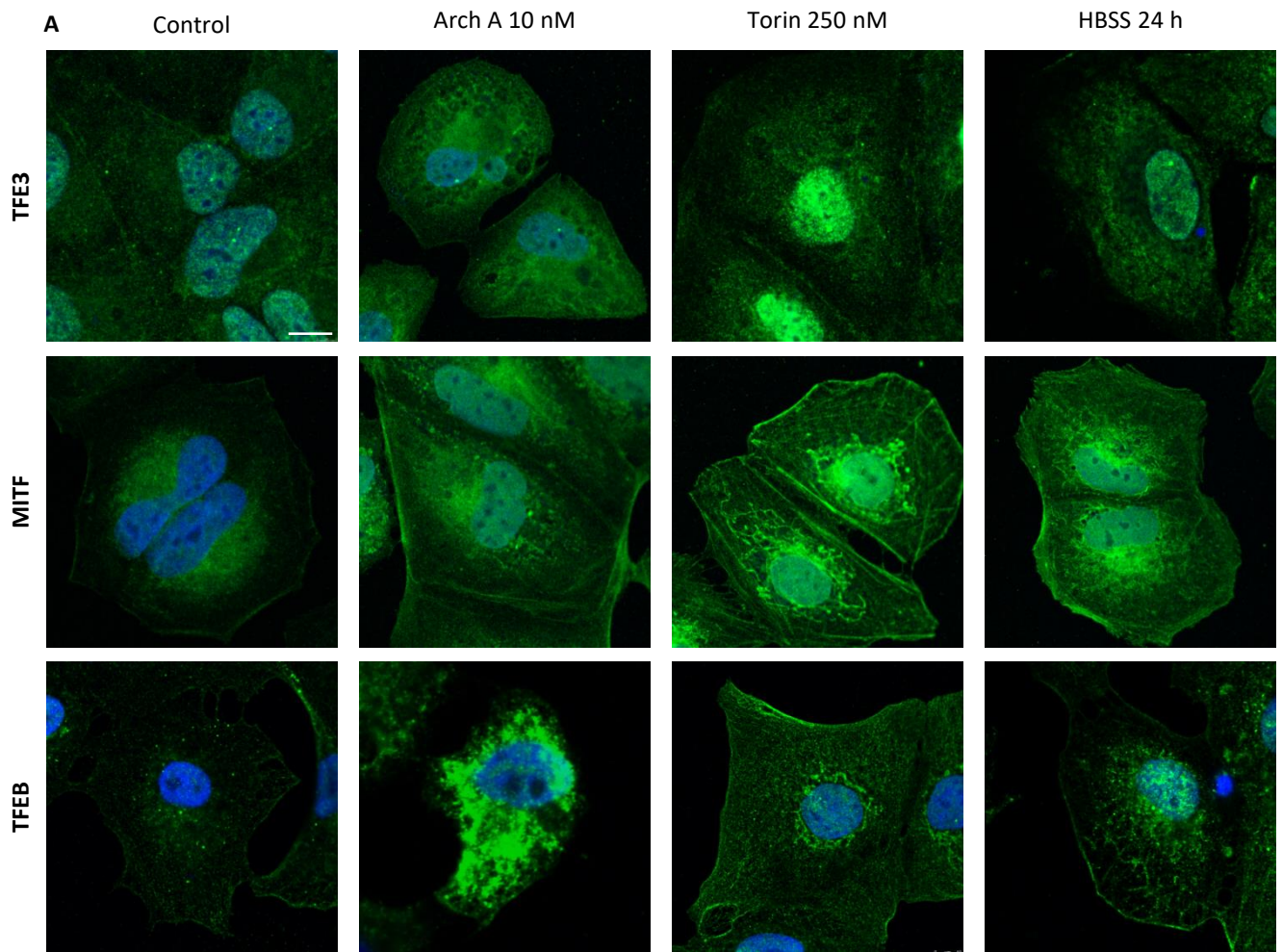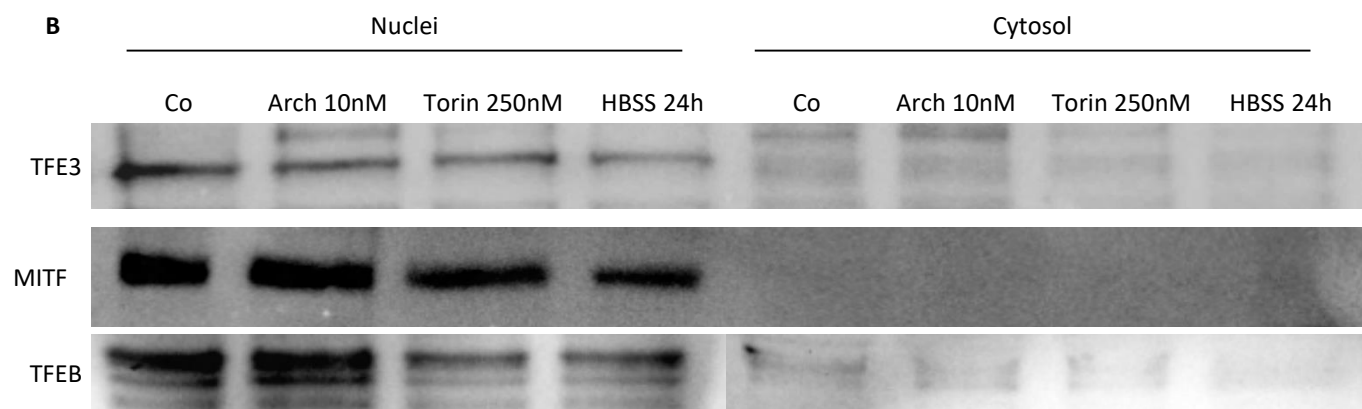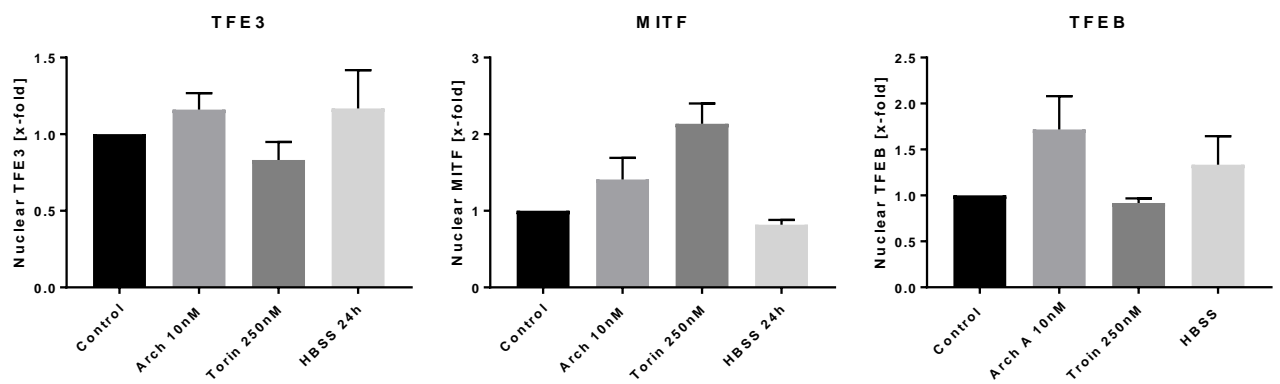

C

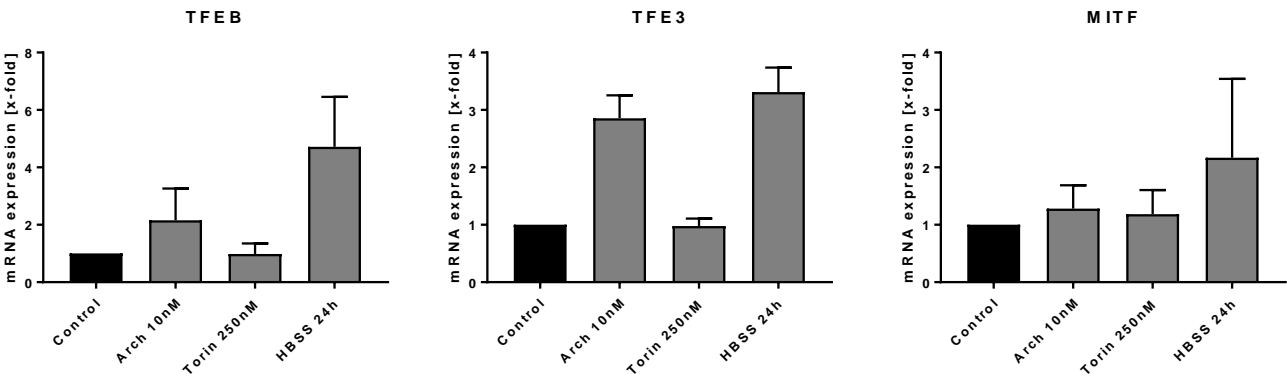

D

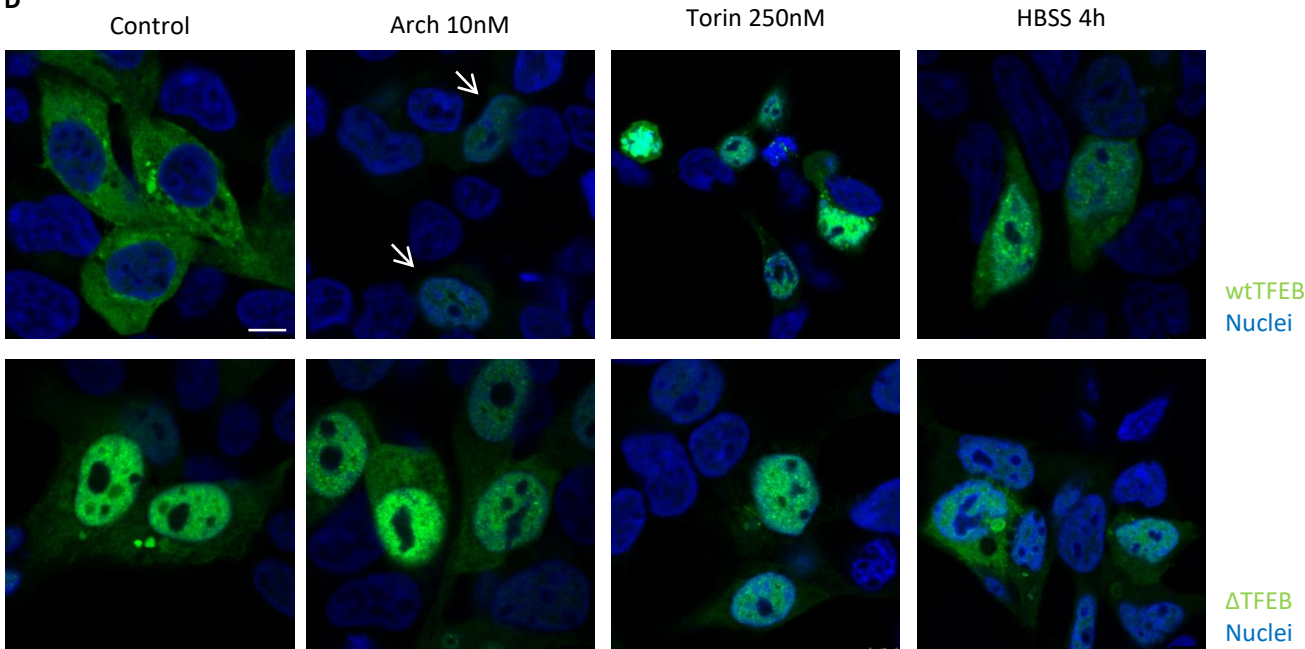

Supplement: Supplementary file 5 — Figure S5. HUH-7 cells were treated as indicated. Representative images out of three independent experiments are shown. (A) Cells were fixed and stained for TFE3, MITF or TFEB, respectively (green) and nuclei (blue) and analyzed by confocal microscopy. Scale bar 10 μm. (B) Cell lysates were fractioned in nuclei and cytosolic fractions and was protein expression of TFE3, MITF and TFEB, was detected by WB. Total protein served as loading control. Nuclear levels of transcription factors were quantified (bar graphs) (C) Relative mRNA expression levels of TFEB, TFE3 and MITF were detected by qPCR. (D) Wilde-type TFEB and a consitutively active TFEB mutant (TFEB) tagged with GFP (green) were overexpressed in HEK 293 cells. Cells were fixed, co-stained for nuclei and analyzed by confocal microscopy. Scale bar 7.5 μm. (PDF 673 kb) [file 12964_2019_399_MOESM5_ESM.pdf]
